# Treatment of nasopharyngeal carcinoma with pulmonary tuberculosis and gout: A case report

**DOI:** 10.3892/ol.2014.2180

**Published:** 2014-05-26

**Authors:** LU WU, MEI LI, DINGYI LIU, MING JIANG, YANYANG LIU, ZHIXI LI, XIA WANG, YANG YU, FENG LUO

**Affiliations:** 1 Department of Medical Oncology, Cancer Center and State Key Laboratory of Biotherapy, West China Hospital of Sichuan University, Chengdu, Sichuan 610041, P.R. China; 2 Department of Oncology, Chengdu Seventh People’s Hospital, Chengdu, Sichuan 610041, P.R. China

**Keywords:** combination treatment, gout, nasopharyngeal carcinoma, pulmonary tuberculosis

## Abstract

In China, the incidence of nasopharyngeal carcinoma (NPC) and tuberculosis remains high. Additionally, there has been a marked increase in the prevalence of gout. In recent years, there has been an increase in the number of co-existing diseases. To the best of our knowledge, there have been no previous cases reported in the literature with regard to patients suffering from NPC complicated with pulmonary tuberculosis and gout. The present study describes the case of a 59-year-old male with this condition. The patient received a combination of anti-tumor, anti-tuberculosis and anti-gout therapies, and experienced no severe adverse reactions during treatment. At present, the patient’s Eastern Cooperative Oncology Group performance status is good, there has been no local recurrence or distant metastasis of the NPC, and the pulmonary tuberculosis and gout are well controlled. The aim of this study was to provide insight into the treatment of patients suffering from co-existing conditions.

## Introduction

The incidence of nasopharyngeal carcinoma (NPC) varies by region and ethnicity, with the highest incidence in Southeast China and Southeast Asia (1,2). Currently, the leading treatment for NPC is radiotherapy. In addition, a combination of radiotherapy and chemotherapy can improve the prognosis of patients with advanced NPC. There are numerous therapeutic strategies for combining radiotherapy with chemotherapy, and the optimal regimen continues to be explored. In recent years, epidemiological evidence has shown that the tuberculosis epidemic situation in China is not optimistic, and although the incidence has declined, the level remains high (3,4). In addition, there has been a marked increase in the prevalence of gout, which highly correlates with economic development as manifested by dietary and lifestyle changes (5,6). To the best of our knowledge, there have been no previous cases reported with regard to patients with NPC complicated with pulmonary tuberculosis and gout. The current study presents the case of a patient who was successfully treated for this condition. Patient provided written informed consent.

## Case report

**Figure 1:**
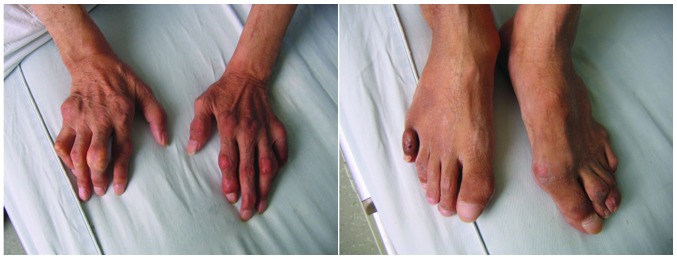
Images of the tophi. Multiple tophi were found on the hands and feet.

**Figure 2:**
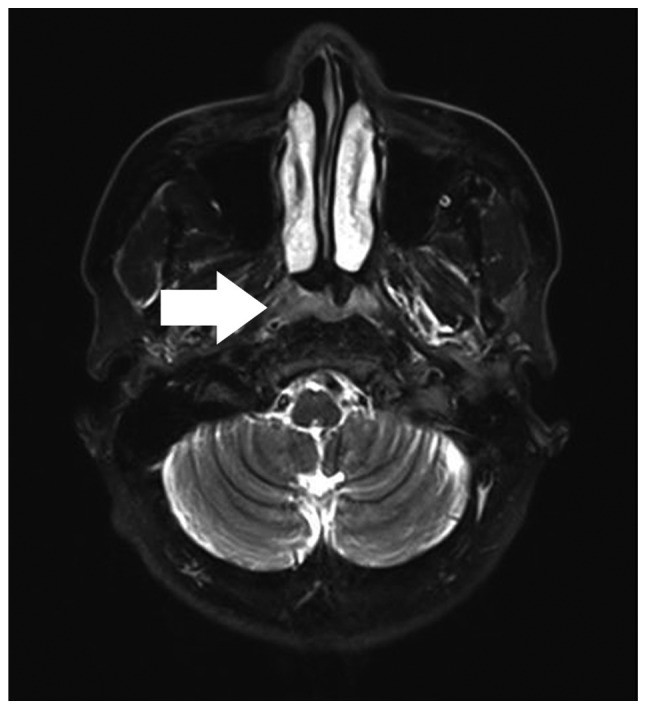
Initial magnetic resonance imaging (MRI) scan of the nasopharynx showing soft-tissue thickening of the top wall of the nasopharynx (arrow).

**Figure 3:**
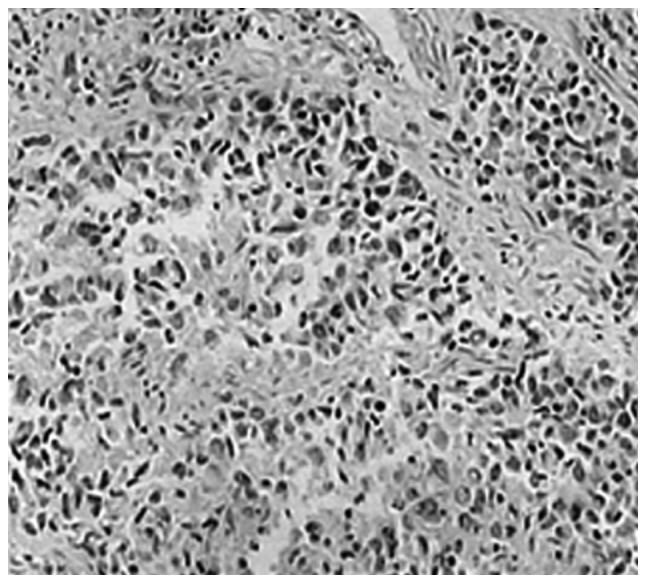
Microscopic features of the tumor. The main tumor consisted of poorly-differentiated squamous cells (hematoxylin and eosin staining; magnification, ×400).

**Figure 4:**
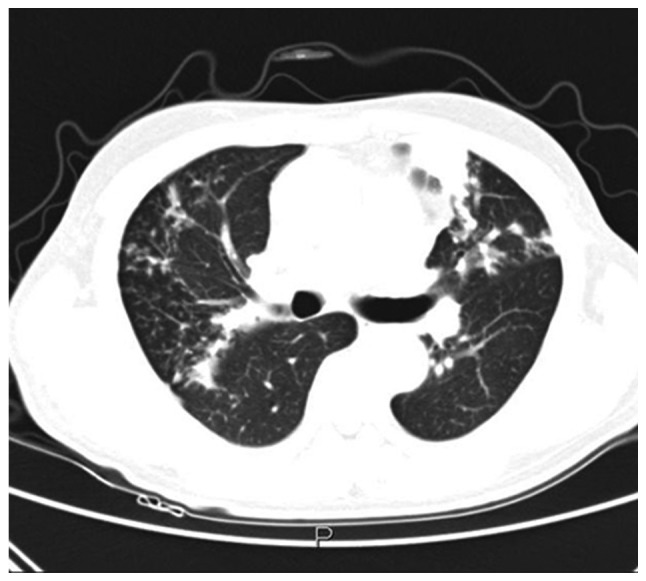
Initial Computed tomography (CT) scan of the lungs showing increased lung markings, plaques and nodules of varying sizes.

**Figure 5:**
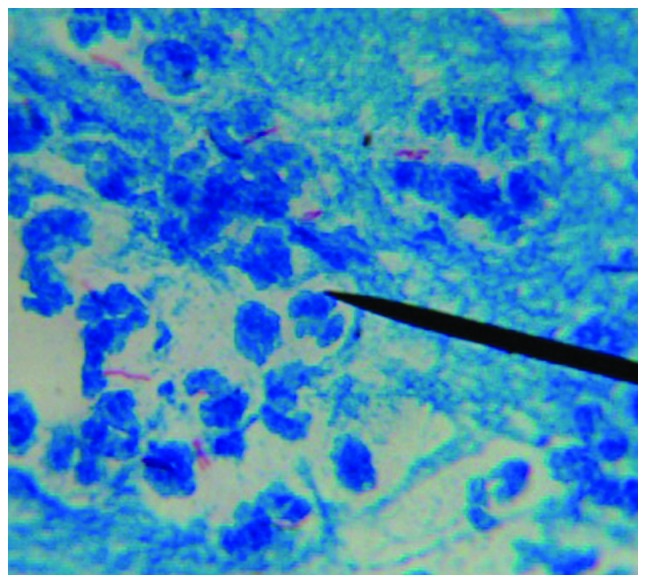
Microscopic features of the sputum smear. Acid fast bacilli dyed red (acid-fast stain; magnification, ×1,000).

On February 21, 2011, a 59-year-old male was admitted to the West China Hospital of Sichuan University (Chengdu, China) with a 5-month history of blood in the nasal mucus. Five months prior to admission, the patient intermittently experienced a runny nose with a small amount of dark red blood, which was occasionally accompanied by dizziness. A physical examination revealed a rubbery and fixed lymph node on the right side of the neck, which was 1 cm in diameter. Multiple tophi were found on the hands and feet (Fig. 1). The patient had been suffering from gout for 20 years, and had no history of tuberculosis. Magnetic resonance imaging (MRI) of the nasopharynx showed soft-tissue thickening of the top wall of the nasopharynx (Fig. 2). A nasopharyngeal fiberoptic laryngoscope examination revealed a nasopharyngeal neoplasm. Moreover, histopathological examination of the nasopharynx revealed poorly-differentiated squamous cell carcinoma (Fig. 3). The contrast-enhanced computed tomography (CT) chest scan and thin-slice high-resolution CT findings indicated secondary tuberculosis, as manifested by increased lung markings, plaques and nodules of varying sizes (Fig. 4). The tuberculosis antibody test was positive, and a sputum smear examination revealed acid-fast bacilli (Fig. 5). Biochemical examination showed uric acid levels of 610 mmol/l.

Upon admission, the patient was diagnosed with poorly-differentiated T2N2M0 squamous cell carcinoma of the nasopharynx, secondary pulmonary tuberculosis (type III), and gout. Following admission, the patient received anti-tumor, anti-tuberculosis and anti-gout treatments. The anti-tumor therapy was chemoradiotherapy combined with concurrent targeted therapy. The gross tumor volume of the nasopharynx with visible lymph nodes underwent helical tomotherapy at a dose of 70 Gy in 33 fractions, the high-risk clinical target volume received helical tomotherapy at a dose of 60 Gy in 33 fractions and the low-risk clinical target volume received helical tomotherapy at a dose of 56 Gy in 33 fractions. At the same time, cisplatin and nimotuzumab were administered intravenously (i.v.) at weekly doses of 40 mg/m^2^ and 200 mg, respectively. One month after the end of concurrent chemoradiotherapy, the patient received chemotherapy combined with concurrent targeted therapy for four cycles: i.v. infusion of 240 mg liposomal paclitaxel on day 1, i.v. injection of 200 mg oxaliplatin starting on day 1, and every 3 weeks thereafter, and i.v. infusion of 200 mg nimotuzumab weekly. Anti-tuberculosis treatment was according to the regimen: Once-daily (q.d.) administration of 300 mg isoniazid, twice a week administration of 600 mg rifapentine, and q.d. administration of 750 mg ethambutol, which was continued for 6 months. In addition, the patient continued to receive 50 mg benzbromarone daily to treat the gout.

**Figure 6:**
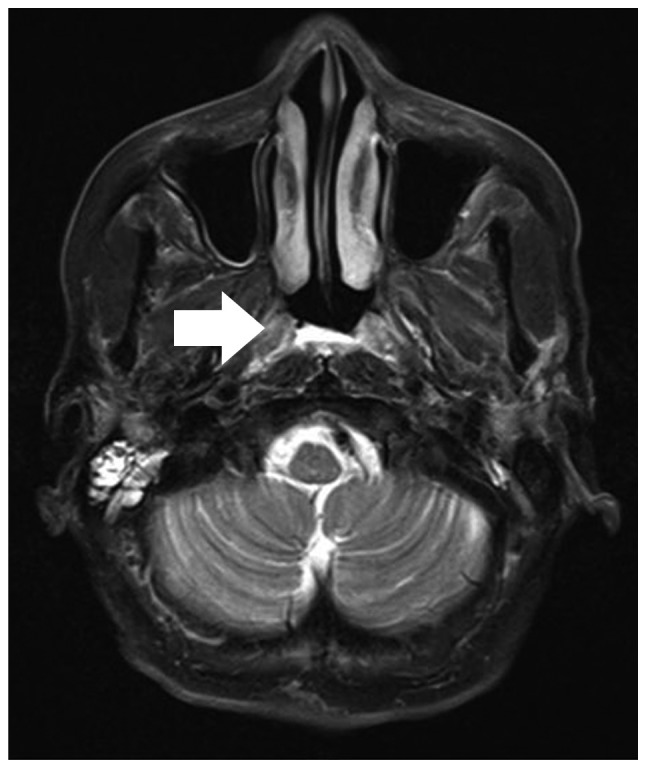
Magentic resonance imaging (MRI) at one month post-anti-tumor therapy (September, 2011) showing a decrease in the soft-tissue thickening at the roof of the nasopharynx (arrow) (compared with results from the MRI performed in February 2011).

**Figure 7:**
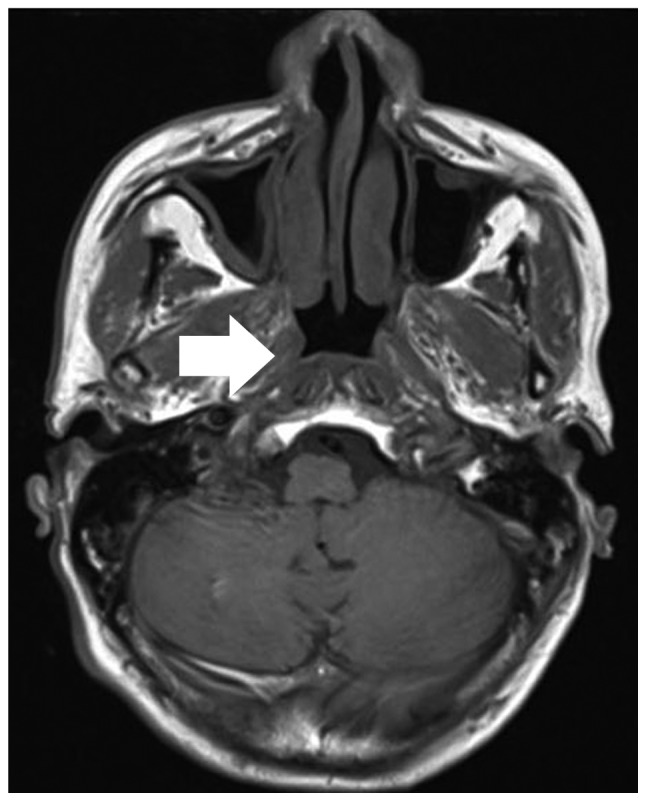
Magentic resonance imaging (MRI) at 11 months post anti-tumor therapy (July, 2012) revealing no evident thickening of the nasopharyngeal wall (arrow).

**Figure 8:**
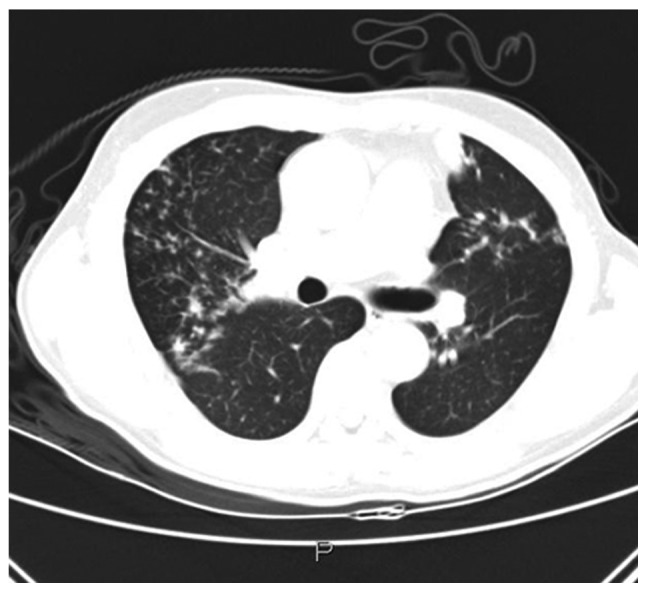
Contrast-enhanced computed tomography (CT) chest scan at two months after the initiation of anti-tuberculosis therapy (April, 2011) demonstarting a decrease in the number of nodules and intrapulmonary patches of varying sizes.

**Figure 9:**
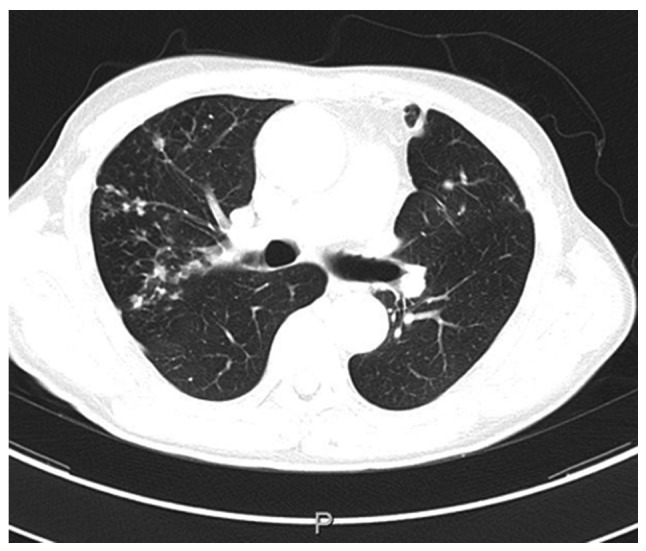
Repeat compted tomography (CT) scans 11 months post-anti-tuberculosis therapy (July, 2012) showing scattered streaks, plaques and nodules throughout the lungs with no obvious changes, which was in contrast with the results from April 2011.

During treatment, the patient maintained normal total, direct and indirect bilirubin levels. The aspartate aminotransferase and alanine aminotransferase levels increased, but returned to normal levels in the late stage of treatment. Although the leukocyte levels in the patient decreased to a minimum of 1.6×10^9^/l during treatment, they quickly increased with symptomatic treatment. Platelet levels fluctuated between normal and ceiling levels, and uric acid levels were mostly maintained at the upper limit of normal. One month after the end of anti-tumor therapy (September, 2011), an MRI showed a decrease in the soft-tissue thickening at the roof of the nasopharynx (compared to results from the MRI taken in February 2011) (Fig. 6). At 11 months post-anti-tumor therapy (July, 2012) another MRI was performed, which did not reveal clear thickening of the nasopharyngeal wall (Fig. 7). Two months after the initiation of the anti-tuberculosis therapy (April, 2011), a contrast-enhanced CT chest scan demonstrated a decrease in the number of nodules and intrapulmonary patches of varying sizes (compared to the results from February, 2011) (Fig. 8). At 11 months post-anti-tuberculosis therapy (July, 2012), repeat CT scans revealed scattered streaks, plaques and nodules throughout the lungs, with no evident changes, which was in contrast to the results from April 2011 (Fig. 9). Multiple sputum smear examinations did not show acid-fast bacilli. Currently, the patient’s Eastern Cooperative Oncology Group performance status is good. There has been no local recurrence and distant metastasis of the NPC, and the pulmonary tuberculosis and gout are under control.

## Discussion

Currently, the National Comprehensive Cancer Network Clinical Practice Guidelines in Oncology recommend concurrent chemoradiotherapy for the treatment of locally advanced NPC (7,8,9). Studies of targeted therapies have been encouraging in recent years. Radiotherapy combined with cetuximab for locally advanced squamous cell carcinoma of the head and neck has been shown to reduce the risk of recurrence and improve survival, without increasing radiation-related adverse effects (10). Recently, an Exercise and Nutritional Intervention for Cardiovascular Health study found that the combined treatment of Erbitux with radiotherapy and chemotherapy for locoregionally advanced NPC was well tolerated, with a local control rate of 100%. Moreover, no local recurrence occurred following a median follow-up time of 330 days, and distant metastasis occurred in only four patients (11). Basavaraj *et al* (12) reported the results of a phase II clinical trial in which 92 patients with advanced head and neck squamous cell carcinoma received standard therapy with or without nimotuzumab, a humanized monoclonal antibody that recognizes domain III of the extracellular region of the epidermal growth factor receptor. The study showed that chemoradiation combined with nimotuzumab conferred a survival advantage (12). In addition, a controlled, double-blind, randomized clinical trial by Rodríguez *et al* (13) showed that patients with advanced squamous cell carcinoma of the head and neck who received a combination of standard therapy and nimotuzumab experienced a clinical benefit, manifested by a median survival time of 12.5 months compared with 9.47 months in patients receiving radiotherapy alone. Moreover, the combined treatment was well tolerated (13). Furthermore, a multi-center phase II clinical trial found that nimotuzumab combined with radiotherapy significantly improved the efficacy of treatment in patients with advanced nasopharyngeal squamous cell carcinoma, with only mild adverse drug reactions (14).

The patient in the present case study had experienced chronic gout for 20 years. Acute uric acid nephropathy easily occurs under the effect of drugs, and can lead to acute renal failure. Chemotherapy, anti-tuberculosis and anti-gout drugs interact in the body, increasing the risk of side-effects. A number of clinical trials have demonstrated that cisplatin can enhance radiosensitivity. Platinum-based chemoradiotherapy has a significant effect in the study of concurrent chemoradiotherapy in NPC (15,16). In addition, platinum drug toxicity and radiotherapy toxicity are not superimposed; however, the optimal dosage of cisplatin to combine with radiation therapy has not been established. Currently, concurrent chemoradiotherapy in patients with locally advanced NPC consists of a high dose of cisplatin (100 mg/m^2^) and radiotherapy. However, certain studies have indicated that low and medium doses of cisplatin have a similar efficacy to high doses of cisplatin, and dividing doses of cisplatin did not reduce the efficacy, but did decrease the toxicity (17,18). In the present case study, a concurrent chemoradiotherapy regimen with 40 mg/m^2^ cisplatin was used, to avoid induction of renal toxicity as uric acid levels increased, while at the same time treating the gout. Oxaliplatin is a third-generation platinum drug that has less toxic side-effects than cisplatin. A previous study postulated that oxaliplatin should be used for the treatment of patients with advanced NPC who are resistant to cisplatin, particularly those with poor renal function (19). The patient in the present study was treated with oxaliplatin in order to avoid increasing the burden on the kidneys. During treatment, the patient maintained good renal function, and the creatinine and urea values remained within the normal range.

According to the 2010 global tuberculosis control report from the World Health Organization (WHO) (20), there are a total of 14 million cases of tuberculosis worldwide. Although the incidence decreases each year, the number of cases continues to increase. The treatment of latent *Mycobacterium tuberculosis* is the cornerstone of tuberculosis elimination. However, the hepatotoxicity of anti-tuberculosis drugs has a great impact on the human body, as it can cause liver damage, and even result in mortality due to liver failure and acute hepatic necrosis. Three randomized controlled trials have shown that the combination of isoniazid and rifapentine treatment for 3 months was more efficacious than the single use of isoniazid for 9 months (21). The combination of isoniazid and rifapentine not only shortened the treatment time, but also enhanced the treatment efficacy and decreased the toxicity (22). Therefore, the patient in the present study received isoniazid, rifapentine and ethambutol to minimize damage to the liver. Although the patient experienced third degree bone marrow suppression, serious complications did not occur during treatment. At present, the patient’s NPC, pulmonary tuberculosis and gout remain under control.

In recent years, there has been an increase in the number of co-existing diseases. When several diseases are being treated at the same time, clinicians must take into account not only the efficacy of the treatment, but also the potential side-effects. Future studies are required on the optimal regimens for treating patients suffering from co-existing conditions.
